# Corneal ulceration following periocular scorpion sting: a case report

**DOI:** 10.1186/s12348-024-00411-3

**Published:** 2024-06-25

**Authors:** Mohammad Shiravani, Mahmood Nejabat, Alireza Attar

**Affiliations:** Department of Ophthalmology, Poostchi Ophthalmology Research Center, School of Medicine, Shiraz University of Medical Sciences, Shiraz, Iran

**Keywords:** Scorpion envenomation, Corneal ulceration, Ocular inflammation, Hemiscorpius lepturus, Pseudomonas infection

## Abstract

**Background:**

Scorpion envenomation, a prevalent medical emergency in rural areas, demands immediate attention due to its potential severity. While ocular manifestations are uncommon, they can lead to significant complications such as corneal ulceration. We present a unique case of corneal ulceration subsequent to a yellow scorpion (Hemiscorpius lepturus) sting near the eye, a scenario not previously documented.

**Case presentation:**

A 34-year-old male sought medical care following a scorpion sting despite prior anti-venom treatment. Clinical examination revealed pronounced ocular inflammation, corneal stromal melting, and anterior chamber inflammation, with microbiological confirmation of Pseudomonas spp infection. Treatment comprised fortified ceftazidime and vancomycin eye drops, alongside topical corticosteroids, leading to visual and corneal healing.

**Conclusion:**

This case highlights the urgency of addressing scorpion envenomation and its potential for severe ocular complications, including corneal ulceration. Prompt diagnosis and targeted therapy with antibiotics and corticosteroids are crucial for favorable outcomes. A comprehensive understanding and timely intervention in scorpion sting-induced ocular manifestations are essential for optimal patient management and outcomes in such cases.

**Supplementary Information:**

The online version contains supplementary material available at 10.1186/s12348-024-00411-3.

## Introduction

Scorpion stings pose an urgent risk, often affecting rural communities; prompt treatment is crucial in this time-sensitive situation [1]. A Comprehensive ocular examination in cases of scorpion envenomation is imperative for formulating efficacious therapeutic interventions. Envenomation may precipitate severe cardiorespiratory compromise, pulmonary edema, and infrequently, cerebral vascular events. Furthermore, coagulation aberrations may stem from venom-mediated effects or perturbations in circulatory dynamics. Rare ocular manifestations encompass transient and cerebral blindness, Bilateral Optic Neuropathy (BON), and branch retinal vein occlusion (BRVO) [2–4].Herein, we present a unique case of corneal ulceration following a scorpion sting in the periocular region. To our knowledge, this represents the first documented instance of a periocular scorpion envenomation resulting in corneal ulceration.

## Case presentation

A 34-year-old male presented to the emergency department of our ophthalmology center with severe ocular symptoms following a recent yellow scorpion (Hemiscorpius lepturus) sting to his left eyebrow. The patient reported experiencing intense ocular pain, sudden vision loss, and periorbital edema. Five days prior to admission, he had received anti-venom treatment immediately following the sting. The patient has not used any native medications during the course of the illness. Confirmation of the scorpion species was obtained through consultation with an entomologist.

**Fig. 1:**
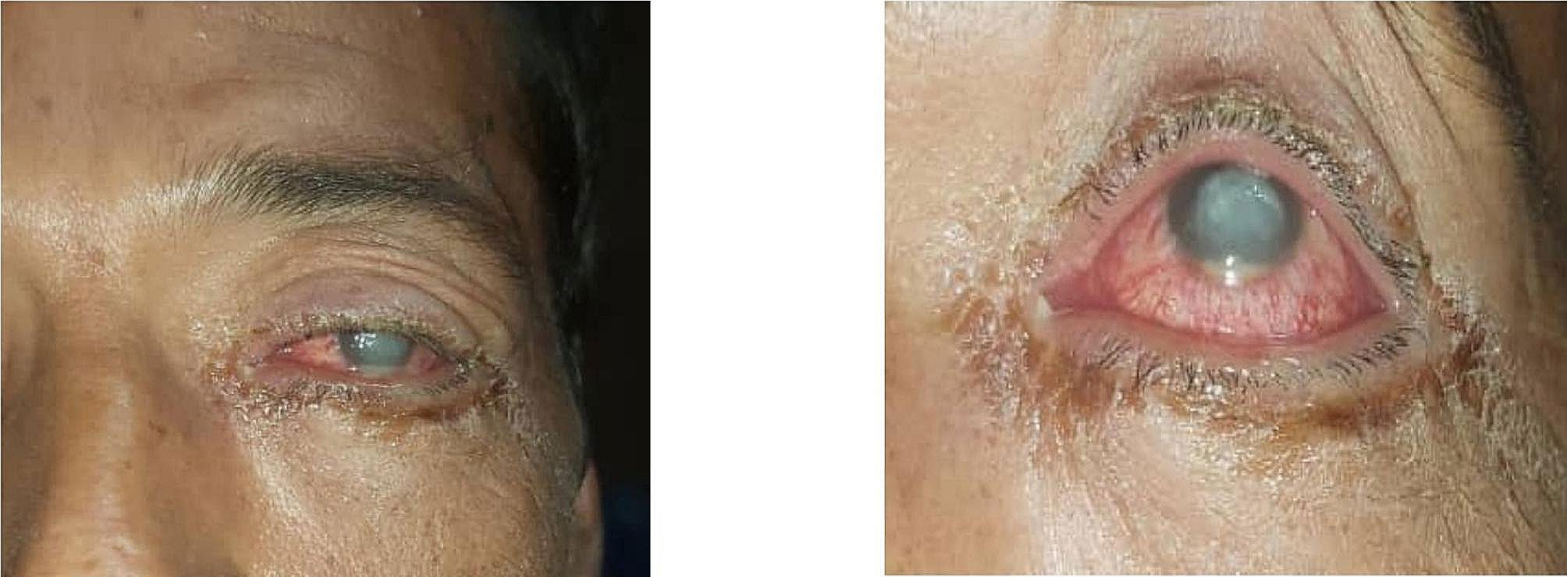
Upon admission, the patient manifests periorbital edema, a 7 × 7 mm corneal infiltration, corneal epithelial defect, and 2 mm hypopyon

Upon initial assessment, the patient’s vital signs were within normal limits, indicating hemodynamic stability. He had no significant medical history of systemic illnesses such as hypertension, diabetes, or coagulative disorders. External examination revealed notable redness, severe upper and lower lid edema, and ptosis at the site of the scorpion bite. The ptosis is classified as ‘mechanical ptosis,’ attributed to tissue induration and edema. Visual acuity assessment demonstrated hand motion perception in the affected left eye and 10/10 vision in the right eye. Notably, no relative afferent pupillary defect was observed, and extraocular muscle function appeared intact. Subsequent slit-lamp examination unveiled severe watery chemosis, indicative of conjunctival inflammation, Additionally, there is gelatinous stromal thinning of 40%, accompanied by whitish infiltration and a corneal epithelial defect at the central cornea, spanning approximately 7 × 7 mm, Additionally, diffuse corneal edema with distinct Descemet membrane folds was noted. Examination of the anterior chamber revealed a normal depth with pronounced flare, substantial cellular infiltration, and a 2 mm mobile hypopyon, indicative of inflammatory response (Fig. 1). Noteworthy, there were no signs of cataract or iris atrophy. Fundus examination was rendered impractical due to severe corneal edema and the extensive anterior chamber cellular reaction. B-scan sonography was employed to evaluate potential involvement of the posterior segment, yielding unremarkable findings.

**Fig. 2:**
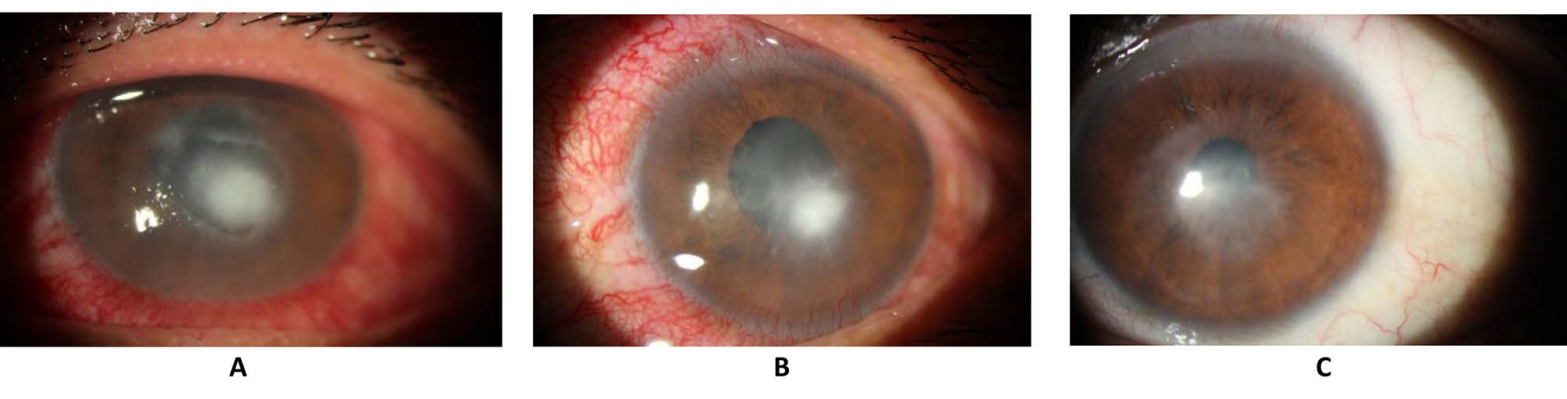
**(A)** depicts the clinical status on the seventh day post-admission, exhibiting prompt improvement following initiation of antibiotic **(B)** illustrates the condition on the tenth day of admission, revealing a reduction to a mere 1 mm corneal infiltration, with a residual central 2 mm x 2 mm epithelial defect **(C)** displays a 4 × 3 mm corneal scar observed on the 60th day following admission

Given the clinical presentation suggestive of infectious keratitis, corneal scrapings were obtained for culture and staining to guide targeted antimicrobial therapy. Broad-spectrum topical antibiotics, including fortified ceftazidime and vancomycin eye drops, were initiated with a loading dose administered every 5 min for 30 min, followed by hourly administration thereafter. Concurrently, topical cycloplegic was prescribed every 8 h to alleviate discomfort and minimize intraocular inflammation. Following the initiation of treatment, a culture conducted on the first day revealed the presence of Pseudomonas spp., which was sensitive to ceftazidime. Subsequently, the antibiotic regimen was adjusted to administer doses every two hours, maintaining the same antibiotics. Over the course of the ensuing days, notable improvements were observed, with reductions in both the size of the corneal epithelial defect and infiltration (Fig. 2A). antibiotics were administered every two hours, which transitioned to every four hours by the third day. By the seventh day, the corneal epithelial defect had decreased to a size of 3 × 3 mm, and the infiltration to 1.5 × 1.5 mm, with the emergence of initial signs of scar formation. Concomitantly, on the seventh day, prednisolone acetate 1% was introduced, to be administered every 12 h. Following the initiation of corticosteroid therapy, a notable facilitation of the recovery process was observed. By the tenth day, a 1 mm corneal infiltration with well-defined borders and a central 2 mm x 2 mm epithelial defect were noted (Fig. 2B). Subsequently, the frequency of prednisolone acetate 1% administration was reduced to every 6 h. On the fourteenth day, the patient was deemed fit for discharge from the ophthalmology ward. Discharge instructions included the continuation of maintenance topical ciprofloxacin, along with a regimen of prednisolone acetate 1% every 6 h.

Upon follow-up evaluation after 2 months, slit-lamp examination revealed a residual central corneal stromal scar measuring 4*3 mm, indicative of prior tissue damage (Fig. 2C). However, the patient had achieved a notable improvement in visual acuity, demonstrating a recovery to 20/60.

## Discussion

Scorpion envenomation incidents are documented throughout diverse Iranian regions, characterized by clinical symptomatology lacking precise determination [5]. The most prevalent clinical manifestations of scorpion bites primarily affect vital organs, including the Central Nervous System (CNS), Cardiovascular System (CVS), respiratory system, coagulation cascade, and endocrine system [2, 5, 6]. Although rare, reports also document ophthalmic manifestations such as blindness resulting from brain infarction leading to cortical blindness, Bilateral Optic Neuropathy, Macular Branch Retinal Vein Occlusion (BRVO), and Transient Ophthalmoplegia [3, 7–9]. Notably, corneal ulceration has been identified as an initial manifestation of scorpion envenomation, representing a novel presentation.

Scorpion venoms are complex mixtures containing salts, free amino acids, peptides, and proteins. They include neurotoxins targeting voltage-gated sodium, calcium, and potassium channels, along with ancillary enzymes like hyaluronidases, metalloproteinases, and phospholipases [10]. Although several studies have proposed that scorpion venom exhibits antibacterial properties that can effectively target specific strains of both gram-positive and gram-negative bacteria [11], in our particular case, the presence of Pseudomonas spp was observed. This finding aligns with the results of the Chinwattanaku study, which reported similar growth patterns of Pseudomonas when exposed to wasp venom [12]. Notably, a distinction exists in our case, as the venom-associated corneal ulcer occurred without the retention of a stinger in the cornea, unlike in instances of wasp venom exposure.Following the initiation of topical corticosteroid therapy, notable enhancements in visual acuity, reduction of ocular inflammation, and accelerated epithelial wound healing were evident. The therapeutic response to prednisolone acetate 1% suggests an immunological reaction to the venom, alongside its efficacy in addressing the infectious process. This effectiveness parallels observations in injuries induced by wasp venom [13], signifying a comparable therapeutic impact on both the immunological and infectious aspects of the condition. In contrast, a study conducted by Gregory et al. elucidated that the utilization of dexamethasone in snake venom-induced injuries did not yield significant improvements in corneal re-epithelialization and was ineffective in mitigating scarring [14]. In conclusion, this case highlights the unique ophthalmic manifestations of scorpion envenomation, including corneal ulceration. Treatment with prednisolone acetate 1% demonstrated efficacy in addressing both the immunological and infectious aspects of the condition.

### Electronic supplementary material

Below is the link to the electronic supplementary material.

Supplementary Material 1

## Data Availability

No datasets were generated or analysed during the current study.
