# Supplementary material for: Corneal ulceration following periocular scorpion sting: a case report
Source: J Ophthalmic Inflamm Infect. 2024 Jun 25;14:30. doi: 10.1186/s12348-024-00411-3 (PMC11199440; doi:10.1186/s12348-024-00411-3)
Supplement: Supplementary file 1 — Supplementary Material 1 [file 12348_2024_411_MOESM1_ESM.pdf]

Academic Ethics Committee in Medical Sciences Research in Shiraz University of Medical Sciences

Informe[illegible] studies

Title of the case report:	Corneal Ulceration Following Periocular Scorpion Sting
Main presenter/supervisor/responsible writer: (name and surname, academic degree, name of the institution, place of work, contact number and address)	Alireza attar – senior resident at Shiraz university of Medical Sciences- department of ophthalmology- 07136291779

.... (legal guardian).................mohammad ozbak...Dear Mr. / Mrs.

By signing this form, you allow Mr. alireza attar to receive information about your illness under the title of Corneal Ulceration Following Periocular Scorpion Sting ... to prepare and present a research plan/article, use the type of case report. Case report designs/articles are usually used to share new information experienced by a patient during the treatment that may be useful for other doctors and members of the treatment group. The results of a case report study may be published in magazines or on the Internet or presented in conferences for information and study by others. This form describes the purpose of this study. Please read it carefully and tell us your opinion, and if you have any questions, don't hesitate to contact me. ..alireza attar .... Do not hesitate.

The purpose of this study is to inform other doctors and members of medical teams that: (Write the main purpose of your study in very simple language without scientific terms in the opposite box)	Corneal ulcer management post-scorpion sting: prompt treatment, antimicrobials, analgesics, .monitoring, collaboration
Information about you/your dependents that is used in this study includes the following and Mr./Mrs. .... (the name of the project manager or study supervisor should be mentioned here) is obliged to preserve your personal information and is not allowed to publish or disclose them (for example, information about your name and family name, date of birth, registration number in the hospital).	Pictures of the patient's eye during corneal .1 ulceration<br>Treatment steps on the patient's eye.2<br>Medicines used for corneal ulceration .3

I assure you that the personal information and identification of you/your dependent in this study will not be published as much as possible and with the greatest confidence, but after the publication of this report, it may be part of the course of actions taken on the disease. You/your dependent will inform others. You will not benefit directly from participating in this study and you will not receive a fee or gift or money, but the information about your disease / your dependent will reach other doctors and members of the treatment

Scanned with CamScanner

teams may be the level of service that In the future, similar patients will be promote By agreeing to provide this case report study, you will not pay, and you will not receive any money or discount. Your participation in this study is optional and you can withdraw from the study whenever you want. In any case, your decision will have no effect on the amount and type of medical services you will receive. You will be informed about any new findings that will be found about your disease / your dependent. Your consent to the implementation of this study means that you have read and agree to the above and you are also allowed to ask about how your/your dependent's disease data will be used at any time. In addition, you agreed that .your information be used in this study

I: ....mohammad ozbak...................... (name and family name of the participant, the patient or his guardian) By signing this form, I confirm that:

1- This case study has been fully explained to me and all my questions and doubts have been fully answered.

2- I am aware of the benefits and risks of this study and I agree with the use of the characteristics of my illness / the illness of my dependent.

3- I was not forced to participate in this study.

4- I have read all the pages of this form.

5- I have given permission to access my/my dependent's personal information.

6- I agree to participate in this study.

date

Name and family name and signature of the participant, the patient or her guardian

...mohammad ozbak................................

........2024/march/1.....

..... Name and signature of the researcher (relevant student/dissertation supervisor)...alireza attar ....

.Shiraz university of medical science department of ophthalmology

:date

2024/march/1

Scanned with CamScanner
